# Prognostic factors for acute ischemic stroke in patients undergoing hemodialysis

**DOI:** 10.1007/s10157-021-02146-0

**Published:** 2021-11-12

**Authors:** Koji Sato, Yusuke Konta, Kyohei Furuta, Kenyu Kamizato, Akiko Furukawa, Akiyuki Ono, Ryo Ogawa, Ryosuke Sato, Kaoru Endo, Tae Yamamoto

**Affiliations:** 1 Division of Internal Medicine, Sendai City Hospital, 1-1-1 Asutonagamachi, Taihaku-ku, Sendai, Miyagi 982-8502 Japan; 2 Division of Neurology, Sendai City Hospital, 1-1-1 Asutonagamachi, Taihaku-ku, Sendai, Miyagi 982-8502 Japan; 3 Department of Clinical Engineering, Sendai City Hospital, 1-1-1 Asutonagamachi, Taihaku-ku, Sendai, Miyagi 982-8502 Japan

**Keywords:** Acute ischemic stroke, Hemodialysis, Dialysis vintage, Intradialytic hypotension, National Institutes of Health Stroke Scale (NIHSS), Modified Rankin scale (mRS)

## Abstract

**Background:**

Acute ischemic stroke (AIS) is a critical complication in patients undergoing dialysis. Although the improvement of AIS management is an urgent requirement, few studies have evaluated the prognostic factors of AIS in these patients. This study aimed to assess the relationship between clinical factors in patients undergoing dialysis and the prognosis of AIS.

**Methods:**

Among 1267 patients who were hospitalized for AIS in Sendai City Hospital from January 2015 to June 2020, 81 patients undergoing hemodialysis were retrospectively enrolled. Multivariate analysis was performed to evaluate the effect of baseline characteristics, dialysis factors, and neurological severity of patients at admission [National Institutes of Health Stroke Scale (NIHSS) score] on in-hospital mortality, physical disability, and the need for rehabilitation transfer.

**Results:**

A higher NIHSS score was a critical risk factor for each outcome and the only significant factor for in-hospital mortality [odds ratio (OR)/point 1.156, 95% confidence interval (CI) 1.054–1.267]. The risk factors of physical disability were NIHSS score (OR/point 1.458, 95% CI 1.064–1.998), older age (OR/year 1.141, 95% CI 1.022–1.274), diabetic nephropathy (OR 7.096, 95% CI 1.066–47.218), and higher premorbid modified Rankin scale (mRS) score (OR/grade 2.144, 95% CI 1.155–3.978); while those of rehabilitation transfer were a higher NIHSS score (OR/point 1.253, 95% CI 1.080–1.455), dialysis vintage (OR/year 1.175, 95% CI 1.024–1.349), and intradialytic hypotension before onset (OR 5.430, 95% CI 1.320–22.338).

**Conclusions:**

Along with neurological severity, dialysis vintage, intradialytic hypotension, and diabetic nephropathy could worsen the prognosis of patients with AIS undergoing hemodialysis.

## Introduction

Cerebrovascular disease is a major cause of death in patients undergoing dialysis due to progressive atherosclerosis and chronic uremia [1]. The frequency of AIS tends to increase compared to cerebral hemorrhage due to aging, increase in the incidence of diabetic nephropathy (DMN), and reduction in the use of anticoagulants due to advances in dialysis equipment [2].

Generally, the risk factors for AIS in patients not undergoing dialysis include age, diabetes mellitus (DM), hypertension, heart failure, smoking history, and history of AIS [3]. Additionally, patients undergoing dialysis are considered to develop cerebral ischemia owing to the elimination of water during dialysis [4]. Furthermore, the brain is vulnerable to a reduction in blood flow because of an impaired autoregulatory capacity due to lower baroreflex sensitivity and reduced peripheral arterial compliance caused by atherosclerosis [5]. Overall, hemodynamic changes during dialysis are important causes of cerebral ischemia, and these changes are partially modifiable by altering the dialysis conditions. The optimal blood pressure to be maintained during daily hemodialysis for the management of AIS is unknown. Moreover, the effect of intradialytic hypotension on the prognosis of AIS has not been clarified.

The risk of atherosclerosis also depends on the etiology of end-stage kidney disease (ESKD), such as DMN in patients undergoing dialysis [6]; moreover, dialysis treatment also leads to atherosclerosis [7, 8]. The impact of hemodialysis patient-specific factors, including ESKD etiology and dialysis vintage (the length of dialysis history), on the prognosis of AIS, remains unclear. We aimed to identify how patients’ clinical characteristics, including dialysis factors, affect the prognosis of AIS in patients undergoing hemodialysis by analyzing their association with mortality, physical disability, and requirement of rehabilitation transfer.

## Materials and methods

### Study patients

Patients who underwent dialysis treatment and were hospitalized at Sendai City Hospital with the diagnosis of AIS between January 2015 and June 2020 were enrolled. Dialysis was avoided for 48 h after onset and performed in a low-efficient manner at least for 2 weeks [9] to avoid exacerbation of neurological symptoms in the initial phase of AIS [10]. Drug administration and rehabilitation were appropriately performed [11].

### Ethical approval

This study was conducted in accordance with the Declaration of Helsinki and approved by the Ethics Committee of Sendai City Hospital (approval number: Senbyousou 591, 20200113). We provided patients with information explaining the purpose and the required individual data for inclusion in the study by posting it on the website of the Sendai City hospital. All patients were provided with the opportunity to opt out.

### Clinical and laboratory parameters

Basic demographics, such as age, gender, body mass index (BMI), and smoking history; and clinical parameters, such as laboratory data, medical history, dialysis factors, types and severity of AIS at admission, treatment of AIS during hospitalization, and outcome at discharge were collected from the electronic medical records. Data concerning history of DM, hypertension, dyslipidemia, atrial fibrillation, ischemic heart disease (IHD), and AIS were collected. Predialysis laboratory data before the onset of AIS, including hemoglobin (Hb) level and serum levels of total protein (TP), albumin (Alb), calcium corrected for Alb (cCa), and phosphorus (P) were also obtained. Dialysis-related factors, including the etiology of ESKD, dialysis vintage, dose of erythropoiesis-stimulating agents (ESA), and the incidence of intradialytic hypotension before the onset of AIS, were collected. Intradialytic hypotension was defined as a reduction in the systolic blood pressure below 100 mmHg or the administration of vasopressor agents.

### Diagnosis of AIS and determination of neurological severity

The diagnosis and classification of AIS and the determination of neurological severity were performed by more than one neurologist. AIS was classified into four categories: cardioembolic stroke (CES), atherothrombotic brain infarction (ATBI), lacunar infarction, and others. The diagnosis of AIS was made by magnetic resonance imaging (MRI). When MRI was contraindicated, computed tomography (CT) was used. If the MRI and CT findings were faint or negative, the clinical diagnosis was made appropriately by neurological symptoms and exclusion of other diseases.

The neurological severity of AIS was evaluated using the NIHSS score (0–42) and the degree of independence in daily living before and after onset was evaluated using the modified Rankin scale (mRS) (0–6), both of which are widely used [12, 13]. The mRS consists of 7 grades rated as follows: 0, no symptoms at all; 1, no obvious disability: despite symptoms, able to perform daily duties and activities; 2, slight disability: unable to do all previous activities, but able to look after own affairs without assistance; 3, moderate disability: requiring some assistance, but able to walk without assistance; 4, moderately severe disability: requiring assistance for walking and physical demands; 5, severe disability: bedridden, incontinent, and requiring constant assistance and attention; and 6, death [13].

### Study outcomes

The outcomes of this study were in-hospital death as survival prognosis, high mRS score (3–5) at discharge as physical disability after AIS, requirement of rehabilitation transfer, and composite outcome of all three events.

### Statistical analyses

The continuous variables were represented by the mean and standard deviation, or median and interquartile range (IQR). The categorical variables were represented by numbers and percentages. Comparisons between groups were conducted using one-way analysis of variance with Tukey–Kramer test, Mann–Whitney *U* test, or *χ*^2^ test as appropriate. Logistic regression analysis was performed to evaluate the association between clinical parameters and each outcome. ESKD etiology was divided into DMN and others, and the classification of AIS was divided into CES and others, because of their frequency and strong effect on patient prognosis. For each outcome, multivariate analyses were performed to reveal prognostic factors. Age, dialysis vintage, intradialytic hypotension before onset, DMN as ESKD etiology, premorbid mRS score, NIHSS score at admission, and significant factors in univariate analysis were assessed in multivariate analysis because of their clinical importance. The degree of association with the outcome was expressed using odds ratios (OR) and 95% confidence intervals (CI). Statistical significance was set at a *P*-value of < 0.05. Receiver operating characteristic (ROC) curves for the association with outcomes based on the analyses were also constructed. All statistical analyses were performed using JMP Pro 14.0.0 (SAS Institute Inc. Cary, USA).

## Results

### Baseline characteristics

From January 2015 to June 2020, 1,267 patients were admitted to Sendai City Hospital with the diagnosis of AIS. Of these, 81 patients undergoing dialysis were enrolled in this study. All of them were undergoing hemodialysis. Table 1 shows the baseline characteristics of the 81 patients. The mean age was 71.1 ± 10.2 years, 54 (66.7%) of the patients were male, and the mean BMI was 22.2 ± 4.5 kg/m^2^. In total, 42 (51.9%) patients had a smoking history. Regarding comorbidities, DM, hypertension, dyslipidemia, atrial fibrillation, and IHD were present in 50 (61.7%), 69 (85.2%), 31 (38.3%), 40 (49.4%), and 13 (16.0%) patients, respectively. Overall, 30 (37.0%) patients had a history of AIS. The most common cause of ESKD was DMN (45 [55.6%]), followed by nephrosclerosis, glomerulonephritis, and autosomal dominant polycystic kidney disease (15 [18.5%], 12 [14.8%], and 4 [4.9%], respectively). The remaining five [6.2%] patients comprised two cases of tubulointerstitial nephritis, one case of lupus nephritis, and two cases of unknown etiologies. The mean dialysis vintage was 7.42 ± 0.63 years. The median premorbid mRS score was 1 [IQR 0, 3], and 58 [71.6%] patients had an mRS score of 0–2. In maintenance dialysis, 33 [40.7%] patients experienced hypotension during dialysis at least once in the previous week before admission. The laboratory findings at the start of hemodialysis were as follows: Hb: 10.9 ± 1.1 g/dL, TP: 6.5 ± 0.6 g/dL, Alb: 3.4 ± 0.4 g/dL, cCa: 9.2 ± 0.8 mg/dL, and P: 5.3 ± 1.5 mg/dL. The median dose of ESA (darbepoetin alfa) was 25 [IQR 15, 45] µg/week.

**Table 1:** Baseline characteristics of 81 patients

Characteristics	Number	%
Age (years)	71.1 ± 10.2	
Dialysis vintage (years)	7.42 ± 0.63	
BMI	22.2 ± 4.5	
Male gender	54	66.7
Smoking history	42	51.9
Intradialytic hypotension	33	40.7
Cause of ESKD		
Diabetic nephropathy	45	55.6
Nephrosclerosis	15	18.5
Glomerulonephritis	12	14.8
ADPKD	4	4.9
Others	5	6.2
Diabetes mellitus	50	61.7
Hypertension	69	85.2
Dyslipidemia	31	38.3
Atrial fibrillation	40	49.4
Ischemic heart disease	13	16.0
History of AIS	30	37.0
Premorbid mRS score (median)	1 (0, 3)	
0	39	48.1
1	8	9.9
2	11	13.6
3	16	19.8
4	7	8.6
5	0	0
6	0	0
Predialysis laboratory data (mean)		
Hb (g/dl)	10.9 ± 1.1	
TP (g/dl)	6.5 ± 0.6	
Alb (g/dl)	3.4 ± 0.4	
cCa (mg/dl)	9.2 ± 0.8	
P (mg/dl)	5.3 ± 1.5	
Dose of ESA (median, mg/week)	25 (15, 45)	

The data are expressed as the mean and standard deviation, or median with 25th and 75th percentiles, or number and %

*ESKD* end stage kidney disease, *ADPKD* autosomal dominant polycystic kidney disease, *AIS* acute ischemic stroke, *Hb* hemoglobin, *TP* total protein, *Alb* albumin, *cCa* calcium corrected for albumin level, *P* phosphorus, *ESA* erythropoiesis-stimulating agents

### Classification of AIS, neurological severity, and patient outcomes

The type and severity of AIS and patient outcomes are shown in Table 2. Among the types of AIS, CES was the most common (35 [43.2%]), followed by ATBI, lacunar infarction, and others (6 [7.4%], 20 [24.7%], and 20 [24.7%], respectively). The median NIHSS score was 3 [IQR 1, 5], and 8 [IQR 2, 20] for CES, 2 [IQR 1.75, 11.75] for ATBI, 2 [IQR 1, 5.75] for lacunar infarction, and 2 [IQR 1, 3.75] for others. CES had a significantly higher NIHSS score than that of lacunar infarction (*P* < 0.001) and others (*P* = 0.001). Forty-two [51.9%] and 19 [23.5%] patients received antiplatelet and anticoagulant therapies, respectively, including continuous treatment. Five [6.2%] patients received intravenous tissue plasminogen activator thrombolysis or intravenous thrombectomy because of early-onset and severe symptoms. Their NIHSS scores were 14, 18, 21, 23, and 26, respectively, and the outcome was death for 80.0% (*n* = 4/5) of these patients. The mean duration of hospital stay was 23.8 ± 16.4 days and the median mRS score at discharge was 3 [IQR 1, 5]. The outcomes at discharge were as follows: 39 [48.1%] continued maintenance dialysis at the original facility, 30 [37.0%] underwent rehabilitation transfer, and 12 [14.8%] died. The cause of death was AIS in seven cases, infectious disease in two cases, heart failure in two cases, and unknown in one case.

**Table 2:** Type and severity of AIS, treatment, and patients outcomes

	Number	%
Classification of AIS		
Cardioembolic stroke	35	43.2
Atherothrombotic brain infarction	6	7.4
Lacunar infarction	20	24.7
Others	20	24.7
Japan Coma Scale (JCS)	0 (0, 3)	
NIHSS score (median)	3 (1, 9)	
0–4	45	55.6
5–9	17	21.0
10–14	4	4.9
15–19	6	7.4
> 20	9	11.1
Treatments		
Antiplatelet agents	42	51.9
Anticoagulant agents	19	23.5
tPA	4	4.9
Endovascular thrombectomy	1	1.2
Length of hospital stay (mean, days)	23.8 ± 16.4	
mRS score at discharge (median)	3 (1, 5)	
0	10	12.3
1	11	13.6
2	6	7.4
3	16	19.8
4	16	19.8
5	10	12.3
6	12	14.8
Outcome		
Return to the original dialysis facility	39	48.1
Rehabilitation transfer	30	37.0
Death	12	14.8

The data are expressed as the mean and standard deviation, or median with 25th and 75th percentiles, or number and %

*AIS* acute ischemic stroke, *NIHSS* National Institutes of Health Stroke Scale, *tPA* tissue plasminogen activator, *mRS* modified Rankin scale

### Risk factors for in-hospital mortality, higher mRS score at discharge, rehabilitation transfer, and composite outcome

To investigate the factors influencing the outcome of AIS in patients undergoing hemodialysis, we examined the relationship between their clinical data and each outcome. In univariate analyses, only a higher NIHSS score was significantly associated with in-hospital mortality (Supplementary Table S1). A significant association between NIHSS score and in-hospital mortality persisted after adjustment for other factors (Table 3).

**Table 3:** Multivariate analysis of factors associated with each outcome

	Unadjusted		Adjusted	
	OR (95% CI)	*P* value	OR (95% CI)	*P*-value
In-hospital mortality^†^				
Age (per 1 year)	1.037 (0.972 − 1.107)	0.272	1.000 (0.918–1.090)	0.994
Dialysis vintage (per 1 year)	1.058 (0.959 − 1.168)	0.263	1.110 (0.989–1.245)	0.077
Intradialytic hypotension before onset	1.046 (0.301–3.629)	0.944	0.719 (0.167–3.085)	0.657
DMN as ESKD etiology	0.769 (0.225 − 2.625)	0.675	0.831 (0.196–3.518)	0.801
Premorbid mRS score (per 1 grade)	1.161 (0.768 − 1.755)	0.478	1.081 (0.654–1.788)	0.761
NIHSS score (per 1 point)	1.129 (1.046 − 1.219)	0.002**	1.156 (1.054–1.267)	0.002**
Physical disability (mRS score: 3 ~ 5)^‡^				
Age (per 1 year)	1.104 (1.043 − 1.168)	< 0.001**	1.141 (1.022–1.274)	0.019*
Dialysis vintage (per 1 year)	1.091 (0.982 − 1.213)	0.106	1.177 (0.994–1.394)	0.059
Intradialytic hypotension before onset	3.850 (1.293–11.460)	0.015*	1.987 (0.389–10.140)	0.409
DMN as ESKD etiology	1.067 (0.403 − 2.827)	0.897	7.096 (1.066–47.218)	0.043*
Premorbid mRS score (per 1 grade)	2.102 (1.335 − 3.309)	0.001**	2.144 (1.155–3.978)	0.016*
Dyslipidemia	0.275 (0.099–0.762)	0.013*	0.557 (0.116–2.686)	0.466
Cardioembolic stroke	3.500 (1.176 − 10.414)	0.024*	0.343 (0.047–2.483)	0.289
NIHSS score (per 1 point)	1.393 (1.109 − 1.751)	0.004**	1.458 (1.064–1.998)	0.007**
Rehabilitation transfer^§^				
Age (per 1 year)	1.005 (0.960 − 1.052)	0.842	0.933 (0.863–1.009)	0.084
Dialysis vintage (per 1 year)	1.115 (1.007 − 1.234)	0.037*	1.175 (1.024–1.349)	0.022*
Intradialytic hypotension before onset	3.329 (1.220 − 9.084)	0.019*	5.430 (1.320–22.338)	0.019**
DMN as ESKD etiology	1.286 (0.490 − 3.372)	0.610	2.324 (0.633–8.540)	0.204
Premorbid mRS score (per 1 grade)	1.163 (0.831–1.627)	0.378	1.091 (0.715–1.666)	0.686
NIHSS score (per 1 point)	1.147 (1.040 − 1.264)	0.006**	1.253 (1.080–1.455)	0.003**
Composite outcome of all three events^||^				
Age (per 1 year)	1.095 (1.038–1.156)	0.001**	1.155 (1.029–1.295)	0.014*
Dialysis vintage (per 1 year)	1.066 (0.970–1.172)	0.186	1.180 (1.014–1.373)	0.033*
Intradialytic hypotension before onset	2.948 (1.025–8.484)	0.045*	1.251 (0.232–6.743)	0.794
DMN as ESKD etiology	1.554 (0.602–4.012)	0.362	19.100 (2.134–170.966)	0.008**
Premorbid mRS score (per 1 grade)	2.223 (1.373–3.560)	0.001**	2.849 (1.386–5.856)	0.004**
NIHSS score (per 1 point)	1.406 (1.115–1.772)	< 0.001**	1.555 (1.145–2.112)	0.005**
Atrial fibrillation	2.833 (1.050–7.649)	0.040*	0.495 (0.061–4.032)	0.511
Cardioembolic stroke	3.401 (1.182–9.787)	0.023*	0.684 (0.068–6.908)	0.748

*ESKD* end stage kidney disease, *NIHSS* National Institutes of Health Stroke Scale, *DMN* diabetic nephropathy, *ESA* erythropoiesis-stimulating agents, *mRS* modified Rankin scale

*P < 0.05, **P < 0.01

^†^Adjusted for age, dialysis vintage, intradialytic hypotension, DMN as ESKD etiology, premorbid mRS score, and NIHSS score at admission

^‡^Adjusted for age, dialysis vintage, intradialytic hypotension, DMN as ESKD etiology, premorbid mRS score, dyslipidemia, cardioembolic stroke, and NIHSS score at admission

^§^Adjusted for age, dialysis vintage, intradialytic hypotension, DMN as ESKD etiology, premorbid mRS score, and NIHSS score at admission

^||^Adjusted for age, dialysis vintage, intradialytic hypotension, DMN as ESKD etiology, premorbid mRS score, atrial fibrillation, cardioembolic stroke, and NIHSS score at admission

Subsequently, we investigated the factors that could affect high mRS scores at discharge after AIS. Based on a previous report [14], an mRS score of 3–5 (requiring some assistance in daily life) was defined as physical disability reflecting a poor functional prognosis. In the univariate analysis, older age, intradialytic hypotension, absence of dyslipidemia, higher premorbid mRS score, CES, and higher NIHSS score were correlated with physical disability (Supplementary Table S2). In multivariate analysis, the significance of age, a higher premorbid mRS score, and NIHSS score persisted, and DMN as ESKD etiology was also found to be a significant factor (Table 3). Similar results were obtained in an analysis that included an mRS score of 6 (death) in the outcome (Supplementary Tables S3 and S4).

We also investigated factors affecting the requirement of rehabilitation transfer because it strongly affects the patients’ quality of life (QOL) and reflects an actual decline in the activity of daily living (ADL) as compared to that before onset. As a result, both before and after adjustment with other factors, dialysis vintage, intradialytic hypotension before onset, and a higher NIHSS score were found to be significant predictors of rehabilitation transfer (Table 3, Supplementary Table S5). Additionally, similar results were obtained in the analysis after the inclusion of death in the outcome, in which the outcome was defined as the inability to continue hemodialysis at the original facility (Supplementary Tables S6, S7).

Composite outcomes including all the three aforementioned events were also evaluated. As a result, 56 [69.1%] patients had at least one of the 3 outcomes, and age, dialysis vintage, DMN as ESKD etiology, premorbid mRS, and NIHSS score were significant predictors of composite outcome in multivariate analysis (Table 3, Supplementary Table S8).

Additionally, we examined the association between the timing of AIS onset in the dialysis schedule and each outcome. Last non-onset time was classified into six groups; during dialysis (*n* = 14), within the day after dialysis (*n* = 19), the day after dialysis (*n* = 24), 2 days after dialysis (*n* = 9), 3 days or more after dialysis (*n* = 4), and unknown (*n* = 11). There was no significant difference between these groups in terms of NIHSS score at admission and each outcome.

### Cut-off values of the NIHSS score for the prediction of each outcome

**Fig. 1:**
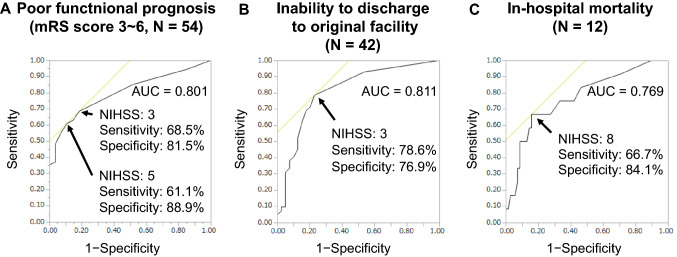
Receiver operating characteristic (ROC) curve for National Institutes of Health Stroke Scale (NIHSS) cut-off values and their association with each outcome. Poor functional prognosis (**A**), inability to discharge to the original facility (**B**), and in-hospital mortality (**C**) are shown. The ROC curves are created for each outcome. The *Y*-axis represents sensitivity and the *X*-axis represents 1 − specificity. The area under the curve (AUC) is expressed for each value. The NIHSS values at which the sum of sensitivity and specificity were maximized are expressed as cut-off values

Since the NIHSS score was found to be a risk factor for all outcomes of AIS in patients undergoing hemodialysis, ROC curves were plotted to better understand their association. While constructing ROC curves, we included mortality in all outcomes. As shown in Fig. 1, the areas under the curve (AUC) of the ROC curve were 0.801, 0.811, 0.769 for physical disability, inability to discharge to the original facility, and in-hospital mortality, respectively, indicating that the NIHSS score is a useful predictor of all three outcomes. Figure 1 also shows the cut-off values for each outcome, which maximizes the sum of sensitivity and specificity, and these values were 3 or 5, 3, and 8 for physical disability, rehabilitation transfer, and in-hospital mortality, respectively.

## Discussion

In this study, AIS in patients undergoing dialysis was found to have a high risk of mortality, and the NIHSS score was a valid predictor of that. Dialysis vintage, DMN as ESKD etiology, and intradialytic hypotension before onset, all of which are specific factors for hemodialysis patients, could predict functional prognosis and rehabilitation transfer. These results could provide useful insights into improving hemodialysis management for the prevention and amelioration of AIS. The overall mortality rate for AIS is 3.7% in Japan [15], and it is reported to be higher (14–15%) in patients undergoing dialysis [16, 17]. Additionally, AIS is often not a direct cause of mortality [16, 17]. Consistently, in this study, the mortality rate of 14.8% (*n* = 12/81) in patients undergoing dialysis was higher than that in patients not undergoing dialysis, and the causes of death in 5 of the 12 patients were complications that developed after admission.

The high risk of mortality for AIS in patients undergoing dialysis is considered to be due to the high incidence of CES and ATBI [16]; atherosclerosis caused by CKD, dialysis, and comorbidities [1, 5, 7, 8]; and the reduction of ADL after initiation of hemodialysis [18–20]. 28.4% of patients in this study had an mRS score of 3–4 before onset, which implies that they required some assistance in daily life. The significant predictor of mortality was solely the NIHSS score, as previously reported [17], and the resulting NIHSS score of 8 could be a useful cut-off value to predict mortality.

The predictors of physical disability assessed by mRS score at discharge were advanced age, higher NIHSS score, higher premorbid mRS score, and DMN as ESKD etiology. Dialysis vintage also tended to be longer in the mRS 3–5 group but was significant in the borderline area.

DMN is an independent risk factor for cardiovascular disease in patients with ESKD [6]. In this study, the average age of patients with DMN was less than that of those without (69.0 years vs. 73.7 years, *P* = 0.039), probably because patients with DMN develop ESKD and cardiovascular events at a younger age. Since DMN and age are inconsistent, it is noteworthy that both independently worsened the functional prognosis. Age was also positively correlated with premorbid mRS score, but not with dialysis vintage in this study population, as previously reported [17, 21].

In summary, age, premorbid mRS score, and DMN, which were expected to be confounding, were all independent risk factors of physical disability. These are important findings because mRS score is a notable prognostic factor of AIS other than mortality; AIS not being often a direct cause of death [16, 17]. As shown in the ROC curve, an NIHSS score of 3 and 5 are useful cut-off values for estimating physical disability with high specificity.

The requirement for rehabilitation transfer is an important outcome in all inpatients with AIS [22]. It is also a serious problem for patients undergoing dialysis, whose average age increases, reflecting ADL decline and dialysis tolerance after admission [18]. The NIHSS score, dialysis vintage, and interestingly, intradialytic hypotension before onset were found as significant risk factors for rehabilitation transfer. Intradialytic hypotension is caused by several factors including cardiac dysfunction [23] and malnutrition [24] and is known to be a risk factor for a lower survival rate in patients undergoing hemodialysis [25]. In this study, intradialytic hypotension and dialysis vintage were not significantly associated with in-hospital mortality or higher mRS score at discharge; however, they were significantly associated with rehabilitation transfer. Intradialytic hypotension increases symptoms associated with dialysis tolerance, such as headache, dizziness, and fatigue [26]. Long-term dialysis induces atherosclerosis, impaired brain autoregulatory capacity [5], and systemic amyloidosis [27]. These conditions, which are not related to neurological findings, are considered to affect actual ADL in patients hospitalized with AIS. The ROC curve showed that an NIHSS score of 3 was a good cut-off value for predicting the need for rehabilitation transfer.

The significant predictors of the composite outcome of all three events were age, dialysis vintage, DMN as ESKD etiology, premorbid mRS, and NIHSS score. These factors were also associated with any other outcome. The result supports the finding that these factors worsen the prognosis and QOL of patients with AIS who were undergoing dialysis.

This study had some limitations. First, this was a single-center retrospective study, and there could be bias or inaccuracies in patient demographics, classification and treatment of AIS, the method chosen for dialysis therapy, and the decision regarding rehabilitation transfer. Second, although disability status at 1 month after the onset of AIS is reported to reliably estimate that after 3 months, which is the standard time point to assess final functional status after AIS [28], mRS score after onset was measured only once at discharge (median 23.8 days after admission). Hence, the long-term prognosis was not evaluated. Third, the frequencies of each outcome were relatively small, and the risk factors for each outcome might not be fully detected. Finally, larger prospective studies are needed to determine how modifying dialysis conditions, such as the prevention of hypotension, actually improves the prognosis after AIS.

## Conclusion

The prevention of AIS is important in patients undergoing hemodialysis with longer dialysis vintage or DMN as ESKD etiology because these are risk factors of worse AIS prognosis, in addition to neurological severity and basal ADL. Prevention of hypotension during hemodialysis enables potential improvement of the prognosis after AIS.
